# Characterization of a novel papillomavirus identified from a whale (*Delphinapterus leucas*) pharyngeal metagenomic library

**DOI:** 10.1186/s12985-023-02009-y

**Published:** 2023-03-20

**Authors:** Xiang Lu, Rong Zhu, Ziyuan Dai

**Affiliations:** 1 Department of Microbiology, School of Medicine, Jiangsu University, Zhenjiang, China; 2 Department of Clinical Laboratory, Affiliated Hospital 6 of Nantong University, Yancheng Third People’s Hospital, Yancheng, Jiangsu China

**Keywords:** Whale, Virome, Papillomavirus, Gene recombination

## Abstract

Here, using viral metagenomic method, a novel whale papillomavirus (temporarily named wPV, GenBank accession number OP856597) was discovered in a whale (*Delphinapterus leucas*) pharyngeal metagenomic library. The complete genome size of wPV is 7179 bp, with GC content of 54.4% and a nucleotide composition of 23.4% A, 22.3% T, 28.4% G, and 25.9% C. The viral genome has a typical papillomavirus organization pattern, and five ORFs were predicted, including two late genes encoding L1 and L2, and three early genes encoding E1, E2, and E6. Pairwise sequence comparison and phylogenetic analysis based on the L1 gene sequence indicated that wPV may be a novel species within genus *Dyodeltapapillomavirus*. In addition, the E2 region of wPV was predicted to have a potential recombination event. The discovery of this novel papillomavirus increases our understanding of the viral ecology of marine mammals, providing insights into possible future infectious diseases.

**Supplementary Information:**

The online version contains supplementary material available at 10.1186/s12985-023-02009-y.

## Introduction

Due to the rapid development and wide application of sequencing technology in recent years, more and more novel viruses have been continuously identified, and the host species population of known viruses has also increased rapidly. Members of the family *Papillomaviridae* are non-enveloped, small, circular viruses with double-stranded (ds) DNA genomes of approximately 5.7 to 8.6 kilobases (kb) in size[1]. Papillomaviruses (PVs) are capable of infecting the skin and mucosal surfaces of mammals and many other vertebrates in a host-specific manner and can cause papilloma or fibromyoidoma in humans, animals, and birds[2, 3]. However, there are several studies that have identified PVs in healthy skin[4] and mucous membranes[5]. At present, numerous identified non-human papillomaviruses (NHPs) have been identified in a wide range of animals including cattle[6], dogs[7], pigs[8], domestic cats[9], manatees[10], giant pandas[11], and cetaceans[12–14], and they are commonly reported to be associated with mucosal and cutaneous lesions.

PVs contain early genes (E1-E8) encoding nonstructural proteins and late genes (L1 and L2) encoding structural proteins, and the open reading frames (ORFs) arrangement of viruses in different animal species is different[15]. Among them, E1, E2 and E4 are regulatory genes involved in transcription and replication, E5, E6 and E7 are potential oncogenes, L1 and L2 are two genes encoding self-assembling proteins that produce viral capsids[16, 17]. There are also some reports that early genes such as E5 and E8 are involved in oncogenesis[18]. According to the International Committee for the Taxonomy of Viruses (ICTV), the family *Papillomaviridae* currently consists of 133 species grouped into 53 genera. Classification of papillomavirus types is now based on the nucleotide sequence of the L1 gene[19]. Members of the same genus of PVs share more than 60% nucleotide sequence identity in the L1 ORF. PV types within a species share 71–89% nucleotide identity in the complete L1 ORF. Sequences that share 90-98% nucleotide identity represent different subtypes within the same type, while sequences that share 98–100% nucleotide identity represent different variants within the same subtype[19–21].

Here, we report the complete genome characterization of a novel whale papillomavirus (temporarily named wPV) identified from a whale (*Delphinapterus leucas*) pharyngeal metagenomic library. The advent of this novel full-length genome adds to our understanding of the diversity of marine mammalian PVs. Our analysis suggests that wPV could be putatively a novel species in the genus *Dyodeltapapillomavirus*.

## Materials and methods

### Metagenome assembly

During the investigation of potential pathogenic viruses in mammals, one available library was downloaded from the SRA database, SRR12366696, uploaded by Du et al.[22], Hainan Medical University, corresponding to the host whale. The sample processing method has been described in the previous[22]. Pfastq-dump v0.1.6 (https://github.com/inutano/pfastq-dump) was used to convert SRA format file to fastq format file, and Bowtie2 v2.4.5[23, 24] was used to remove host sequences. Potential primer sequences of raw reads were trimmed using Trim Galore v0.6.5 (https://www.bioinformatics.babraham.ac.uk/projects/trim_galore) and the resulting files were quality controlled with options ‘--phred33 --length 35 --stringency 3 --fastqc’. Duplicated reads were marked using PRINSEQ-lite v0.20.4 (-derep 1)[25]. This library was assembled in-house pipeline. Single-end reads were assembled with MEGAHIT v1.2.9[26] with default parameters. The results were then imported into Geneious Prime v2022.0.1[27] for sorting and manual confirmation. To reduce false negatives during sequence assembly, further semi-automatic assembly of those contigs or singlets with sequence length < 500 bp was performed and contigs with sequence length > 1500 bp after reassembly were retained, where the individual contig was used as reference for mapping to the raw data using the Low Sensitivity/Fastest parameter in Geneious Prime. In addition, mixed assembly was performed using MEGAHIT in combination with BWA v0.7.17[28] to search for unused reads for possible low abundance contigs.

### Searching for viruses in the whale library

The contigs were aligned with the non-redundant protein (nr) database using blastx program built in DIAMOND v2.0.15[29], with a cut-off E-value of < 10^− 5^. In addition, the sequences of proteins such as RdRp (RNA-directed RNA polymerase), Rep (replication-associated protein) and NS1 (non-structural protein) were also downloaded from the RefSeq database to align contigs with sequence length > 1500 bp. The rma2info program built into MEGAN6[30] was used to perform taxonomic identification. Putative open reading frames (ORFs) were predicted by Geneious Prime with built-in parameters (Minimum size: 100)[27], and were further checked through comparing to related viruses. The annotations of these ORFs were based on comparisons to the Conserved Domain Database (CDD).

### Phylogenetic analysis

To infer phylogenetic relationships, nucleotide and protein sequences of reference strains belonging to different group of corresponding viruses were downloaded from the NCBI GenBank database. Related nucleotide and protein sequences were aligned using alignment program implemented in the CLC Genomics Workbench 10.0 (https://digitalinsights.qiagen.com), and the resulting alignment was further optimized using MUSCLE in MEGA v7.0[31] and MAFFT v7.3.1 employing the E-INS-I algorithm[32]. Sites containing more than 50% gaps were temporarily removed from alignments. Bayesian inference trees were then constructed using MrBayes v3.2[33]. The Markov chain was run for a maximum of 1 million generations, in which every 50 generations were sampled and the first 25% of Markov chain Monte Carlo (mcmc) samples were discarded as burn-in. Maximum Likelihood trees were also constructed to confirm all the Bayesian inference trees using software MEGA v7.0[31]. Colorcoded distance matrix analysis between novel papillomavirus and other members of *Papillomaviridae* were performed with Sequence Demarcation Tool v1.2[34].

### Prediction of potential genome recombination events

Genomic alignments of reference strains and the wPV strain were analyzed using the algorithms (RDP, GENECONV, Chimaera, MaxChi, BootScan, SiScan) of the Recombination Detection Program v4.39 (RDP4) software to screen for potential recombination events[35].

### Prediction of spatial structure

ColabFold[36] was used to predict the three-dimensional structure of the viral structural protein identified in this study, and SWISS-MODEL[37] was used to compare and screen models with similar spatial structures from the PDB database. PyMOL v2.0 (www.pymol.org) was used for visualization.

### Data availability

The novel papillomavirus sequence obtained in this study have been deposited in GenBank database under accession number OP856597.

## Results

### Viral metagenomic overview

This library generated a total of 9,167,717 raw reads on the Illumina HiSeq platform, the number of clean reads obtained after quality control is 9,166,522. After queried of the clean reads against the nr database, a total of 581,875 reads had the best matches with viral proteins (Supplementary Table 1), accounting for 6.35% of the total clean reads. About 17 viral families were detected, the most abundant viral family was *Siphoviridae* (68.27% of the total clean viral reads), followed by *Picobirnaviridae* (13.74%), *Myoviridae* (10.64%), *Papillomaviridae* (3.31%), *Podoviridae* (1.34%), *Inoviridae* (1.04%), *Ackermannviridae* (0.52%), and *Mimiviridae* (0.30%) (Supplementary Table 1). In addition, there are 20,152 reads that have not been clearly assigned to viruses at any taxonomic level (E-value > 10^− 5^) and potentially novel viruses may be present in these reads.

### Identification of a novel papillomavirus

**Fig. 1:**
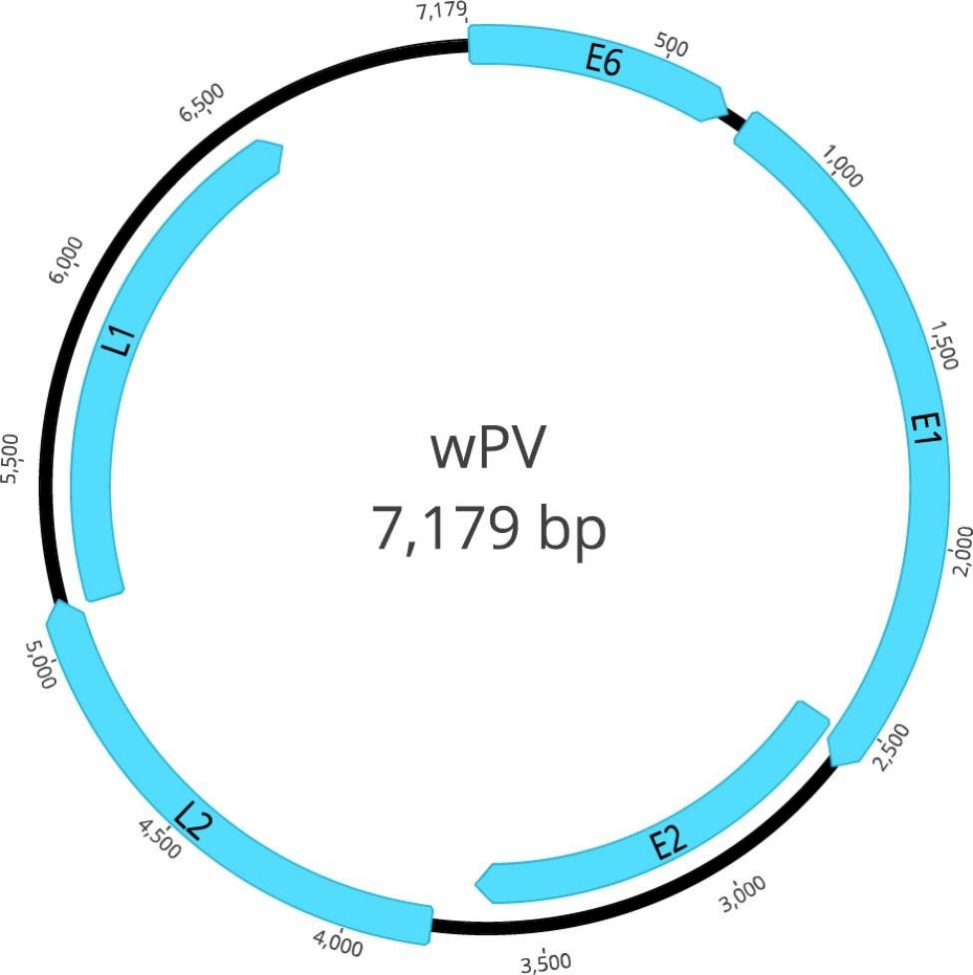
Genomic organization of wPV identified in a whale pharyngeal metagenomic library

In this study, one circular complete genome (temporarily named wPV) related to *Papillomaviridae* was obtained using the assemble sequences program in Geneious Prime v2022.0.1. The complete genome size of wPV is 7179 bp with GC content of 54.4% and a nucleotide composition of 23.4% A, 22.3% T, 28.4% G, and 25.9% C. The characteristic organizational pattern of this complete viral genome is shown in Fig. 1. Five ORFs were well predicted, including two late genes encoding L1 and L2, and three early genes encoding E1, E2 and E6. One ATP-dependent helicase motif (GPPDTGKS; aa 445–452) and five cyclin A interaction motifs (RXL; aa 62–64, aa 108–110, aa 247–249, aa 258–260, aa 505–507) were predicted in the E1 gene. The long control region (LCR) is 569 bp long and is located between the L1 gene and the E6 gene of the circular genome (nt 6611–7179). In LCR region, there are one E1 binding site (E1BS; ATNGTTN_3_AACNAT; nt 7084–7098), three E2 binding sites (E2BS; ACCN_6_GGT; nt 6927–6938, nt 7046–7057, nt 7125–7136) and two nuclear factor 1 binding sites (NF1BS; TTGGC; nt 6726–6730, nt 6886–6890). In the protein encoded by the E6 gene, there are two conserved metal-binding domains (CX_2_C-X_29_-CX_2_C) separated by 36 aa (aa 28–64 and aa 101–137). Furthermore, there is a PDZ-binding motif (XS/TXV/L; YSDL; aa 182–185)[21].

### Phylogenetic analysis

**Fig. 2:**
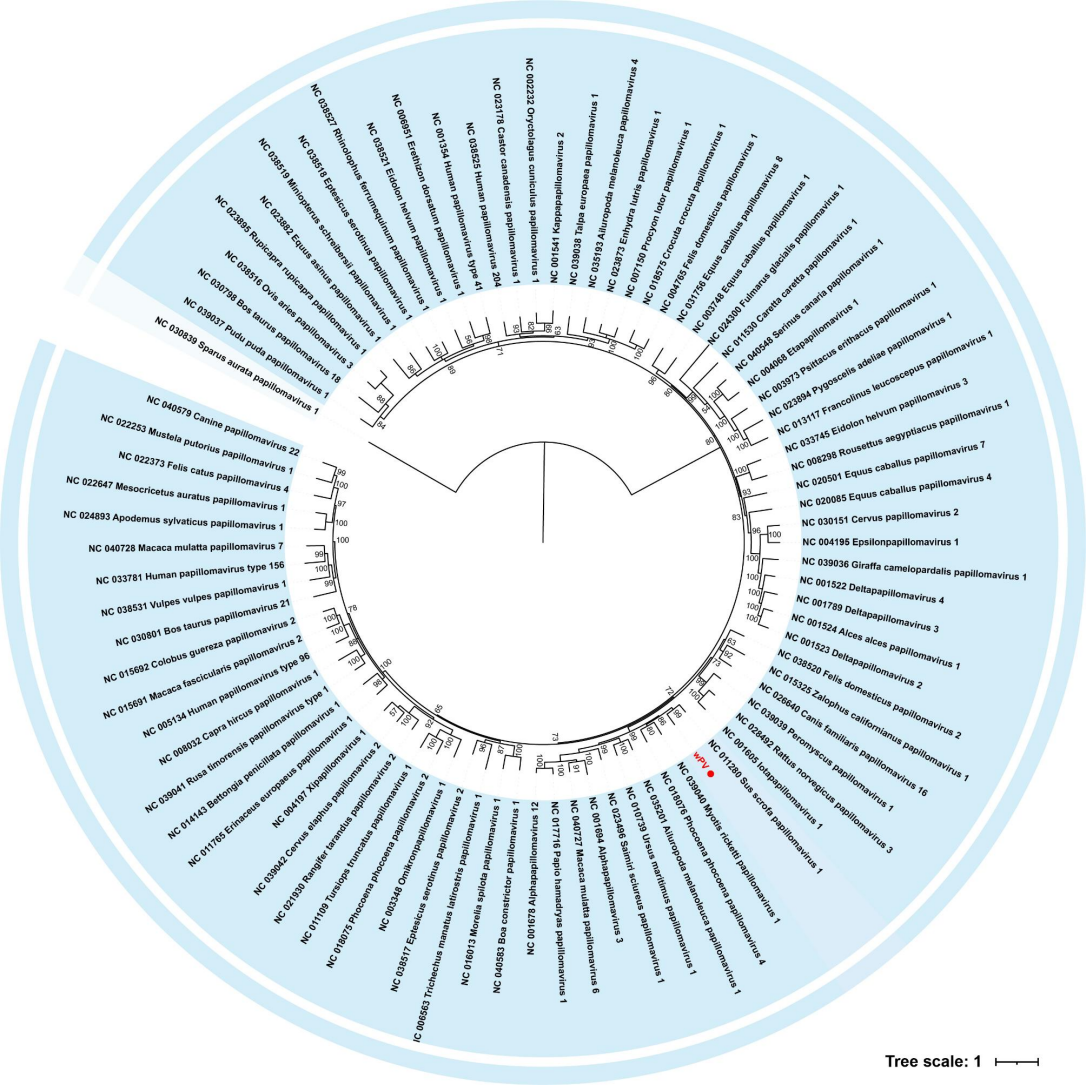
Phylogenetic analysis of wPV. Bayesian inference tree based on nucleotide sequence of the L1 region of virus belonging to *Papillomaviridae* identified here. Within tree the virus found in this study is marked with red

Representative sequences of the current 53 genera of the family *Papillomaviridae* were included in the subsequent analysis, phylogenetic analysis based on the nucleotide sequence of the L1 gene revealed that wPV clustered in a sister clade with Sus scrofa papillomavirus type 1 (NC_011280) belonging to the genus *Dyodeltapapillomavirus* (Fig. 2). Currently, the genus *Dyodeltapapillomavirus* contains only one species, *Dyodeltapapillomavirus 1*.

**Fig. 3:**
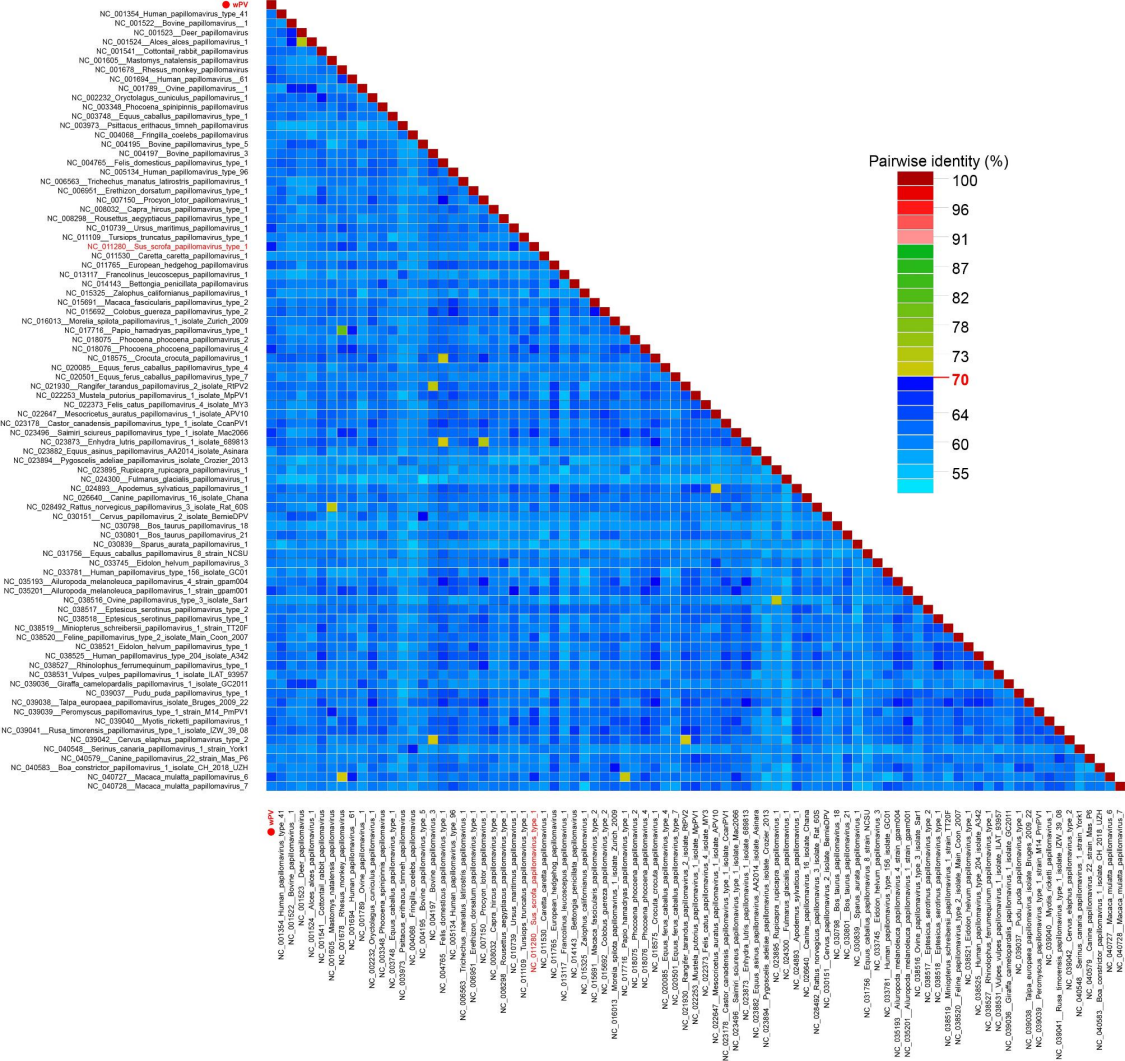
Distance matrix analysis of wPV. Pairwise sequence comparison produced with L1 nucleotide sequences within Bayesian consensus tree

Distance matrix analysis of the L1 gene showed that wPV shared < 70% identity with the nucleotide sequence of Sus scrofa papillomavirus type 1 (Fig. 3 and Supplementary Table 2). According to Taxonomy Guidelines of THE PAPILLOMAVIRUS EPISTEME (PaVE) (https://pave.niaid.nih.gov/explore/taxonomy/taxonomy_concept), wPV should be considered a novel species belonging to the genus *Dyodeltapapillomavirus*.

### Recombination analysis

**Fig. 4:**
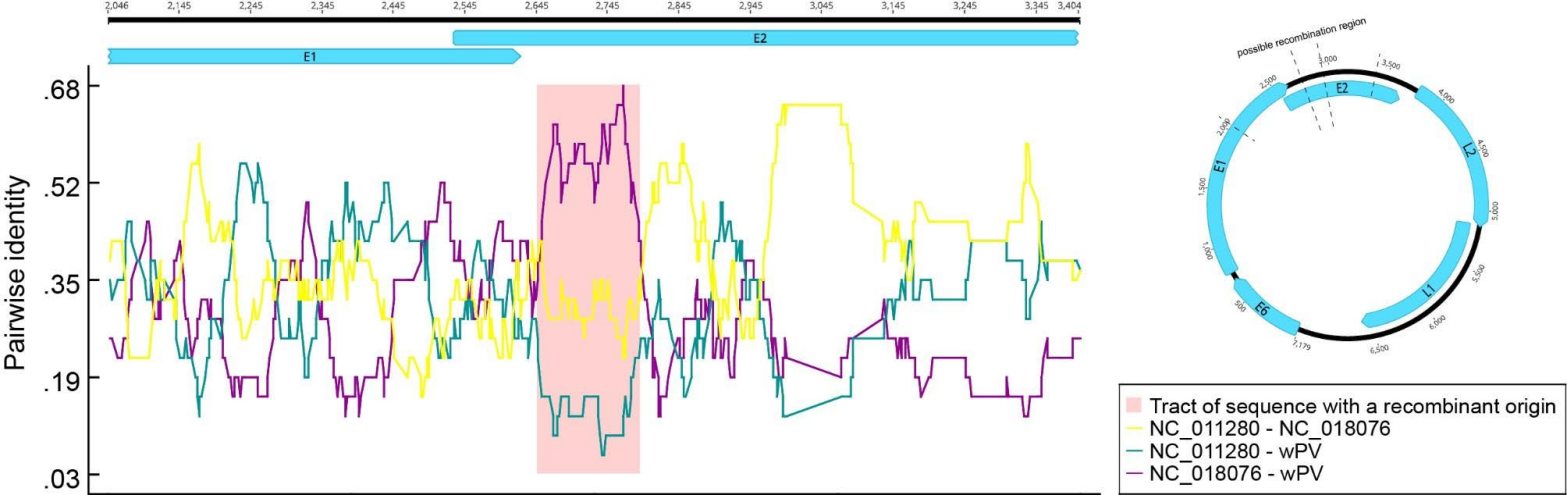
Putative recombination events in the wPV. The region with a pink background represents the potential recombination region

Reference sequences belonging to the family *Papillomaviridae* closest to wPV were screened using blastx program. Using the suite of six recombination detection methods implemented in RDP4, four algorithms (RDP, Chimaera, BootScan, SiScan) supported the detection of recombination signal in the E2 region (wPV; nt 2767–2938), with *P* values of 1.871⋅10^− 3^, 2.678⋅10^− 2^, 4.397⋅10^− 2^, 1.253⋅10^− 4^. The major parent of the wPV was the Sus scrofa papillomavirus 1 (NC_011280; *Dyodeltapapillomavirus*), the minor parent was the Phocoena phocoena papillomavirus 4 (NC_018076; *Dyopipapillomavirus*) (Fig. 4). These two possible parents are both closely related to wPV in the above phylogenetic analysis (Fig. 2).

### Prediction of spatial structure of wPV structural protein L1

**Fig. 5:**
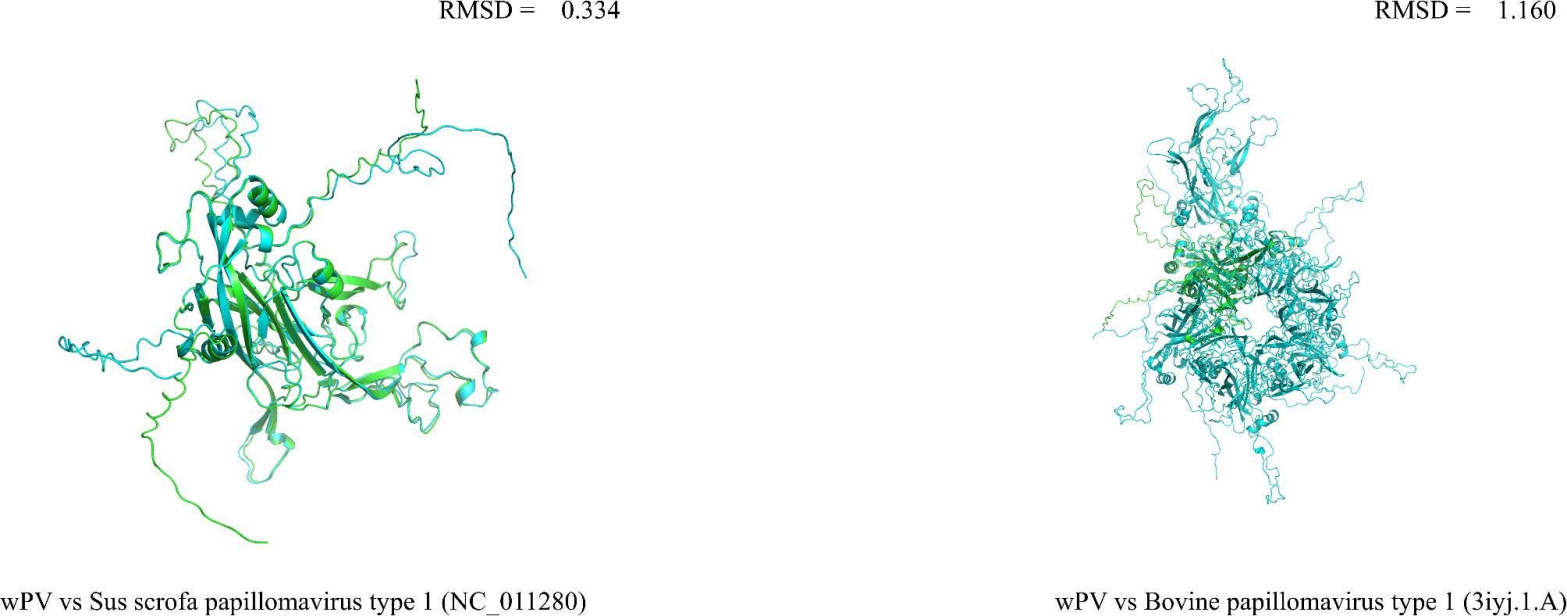
Viral L1 protein structural model visualization. PDB files were visualized and pairwise aligned using PyMOL v2.0 software

The major capsid protein L1 of papillomavirus is a protein of about 55kD that has the ability to spontaneously self-assemble into virus-like particles (VLP). In order to predict and compare the similarity between the spatial structure of wPV structural protein L1 and the structure encoded by the currently known sequences, the sequence with the highest degree of identity to wPV structural protein L1, Sus scrofa papillomavirus type 1 (NC_011280), was downloaded from the GenBank database. ColabFold was used to predict the spatial structure of sequences encoding structural protein L1. In addition, the most similar virus model (Bovine papillomavirus type 1; 3iyj.1.A) to wPV was searched and downloaded in SWISS-MODEL. All PDB files were imported into PyMOL software and do pairwise comparisons, an RMSD of less than about 2 Å would generally be considered very close. In this research, wPV has good similarity with Sus scrofa papillomavirus type 1 and Bovine papillomavirus type 1 in L1 spatial structure (Fig. 5). This suggests that wPV and these viruses may share similar properties in mediating cell attachment during infectious entry.

## Discussion

In recent years, an increasing number of novel viruses are being discovered and characterized in a variety of settings, especially novel PVs of human and animal sources[38], largely due to the rise and advancement of next-generation sequencing, which allows researchers to easily detect viruses on the skin[39] or in vivo[40]. Some of these newly discovered viruses tend to accompany the emergence and progression of disease, but in most cases, these viruses are harmless to humans or animals. Nonetheless, coexistence of viruses with healthy humans and animals does not completely rule out their pathogenic potential. Understanding how these newly identified viruses affect humans or animals will allow us to determine aggressive prevention and treatment strategies.

Members of PVs infect the skin and mucosal surfaces of diverse animal hosts[15], typically resulting in benign mucosal and skin lesions[3]. The core ORFs of papillomavirus include E1, E2, L1 and L2. Previous studies have pointed out that cetacean PVs lack E5 and E7 genes and the E6 protein is larger than other species, and the identification of wPV further supports this view[41, 42]. But we did not predict other ORFs in the wPV genome.

Members of PVs have the characteristics of double-stranded DNA genome stability, species specificity, and close skin or mucous membrane contact as the prerequisite for transmission, which makes it difficult to explain the spread of the virus between species on a global scale. It is argued that this is due to the co-existence and evolution of PVs with the early ancestors of various hosts through a host-linked co-divergence pattern. This suggests that PVs may be one of the oldest and most widespread viral families[15]. But there is also evidence that evolutionary mechanisms such as interspecific transmission, adaptive radiation, recombination, and positive selection have also played an important role in papillomavirus evolution[13]. Evidence for interspecies and intraspecies recombination of PVs has been explained[17, 43–50]. However, to date only cetacean PVs have reported conclusive evidence of specific recombination events[13, 17, 48]. Previous studies have shown that the first possible recombination event in Cetaceans is localized near the end of E2/beginning of L2, and the second possible recombination site is estimated to be within the viral LCR region[48, 51]. In this study, the possible recombination event of wPV occurred at the beginning of the E2 protein. The two parents of wPV predicted in this study are domestic pig and Harbor porpoise. Combined with the above-mentioned characteristics of PVs infection, we cautiously speculate that there may be recombination between domestic pigs and amphibians. However, considering that the signal of recombination is not very obvious, the results of this study cannot be regarded as conclusive evidence for the existence of recombination in wPV.

In conclusion, the identification of wPV increases our knowledge of marine mammalian papillomavirus diversity and provides limited assistance in improving taxonomy. Although there is no direct evidence of the impact of wPV on the host, further dynamic monitoring of it is warranted in the future.

## Electronic supplementary material

Below is the link to the electronic supplementary material.

Supplementary Table 1. Summary of information for a whale pharyngeal metagenomic library.

Supplementary Table 2. Supplementary information for distance matrix analysis of wPV.

## Data Availability

The novel papillomavirus sequence obtained in this study have been deposited in GenBank database under accession number OP856597.
